# Effects of home-based exercise on anxiety, depression, cancer-related fatigue, and quality of life in colorectal cancer patients: a meta-analysis of randomized controlled trials

**DOI:** 10.3389/fonc.2026.1810117

**Published:** 2026-05-07

**Authors:** Jing Yang, Qiao Zeng, Lili Jiang, Jie Yang

**Affiliations:** 1 Colorectal Cancer Center, West China Hospital, Sichuan University, Chengdu, Sichuan, China; 2 Departmentof General Surgery, West China Hospital, Sichuan University, Chengdu, Sichuan, China

**Keywords:** anxiety, cancer-related fatigue, colorectal cancer, depression, home-based exercise, meta-analysis, quality of life

## Abstract

**Background:**

Colorectal cancer (CRC) patients usually experience a range of distressing symptoms such as anxiety, depression, and cancer-related fatigue, which undermine their quality of life. Home-based exercise is considered a complementary intervention with significant potential for managing these symptoms. This study aimed to determine the effects of home-based exercise on anxiety, depression, cancer-related fatigue, and quality of life in CRC patients.

**Methods:**

A comprehensive search was conducted in PubMed, Web of Science, Scopus, Embase, Cochrane Library, CINAHL, CNKI, WanFang, VIP, and CBM, from their inception to February 2026. Randomized controlled trials investigating the effect of home-based exercise on anxiety, depression, cancer-related fatigue, and quality of life among CRC patients were included. The risk bias of the included trials was assessed using the Cochrane Risk of Bias tool 2.0 (ROB 2.0), and the certainty of the evidence for each outcome was subsequently graded according to the Grading of Recommendations, Assessment, Development and Evaluation (GRADE) framework. For quantitative synthesis, a random-effects model was used for all meta-analyses.

**Results:**

A total of 12 randomized controlled trials involving 802 CRC patients were included. The pooled analyses demonstrated that home-based exercise significantly reduced anxiety (SMD = -1.26, 95% CI: -2.24 to -0.29, low certainty evidence), and cancer-related fatigue (SMD = -0.66, 95% CI: -1.14 to -0.18, low certainty evidence), as well as improved quality of life (SMD = 0.64, 95% CI: 0.18 to 1.10, low certainty evidence) compared to usual care. In contrast, no statistically significant effect was observed on depression levels in this population (SMD = -0.76, 95% CI: -1.81 to 0.30, very low certainty evidence).

**Conclusions:**

Our findings demonstrated that home-based exercise may serve as a complementary therapy to reduce anxiety and cancer-related fatigue in CRC patients while improving their quality of life. However, it did not exhibit positive effects in reducing their depression levels. Given the substantial heterogeneity of results and the low certainty of evidence, future studies should focus on improving study design, with particular emphasis on the use of validated and multidimensional outcome measures, to establish a more robust evidence base.

## 1 Introduction

Colorectal cancer (CRC) remains the third most commonly diagnosed malignancy and the second leading cause of cancer-related mortality worldwide (1). An estimated 1.9 million new cases of colorectal cancer were reported worldwide in 2020, with approximately 930000 related deaths (2). By 2040, these figures are expected to rise to 3.2 million new cases and 1.6 million deaths annually (3). While advancements in treatment have significantly improved survival rates, CRC patients generally experience a formidable cluster of disturbing symptoms, including anxiety, depression, cancer-related fatigue, and a consequent decline in health-related quality of life (4–7). These interlinked sequelae not only impose severe distress on individuals but can also impair treatment adherence, hinder functional recovery, and ultimately affect clinical health outcomes (8). Therefore, the effective management of these conditions has emerged as a critical component of comprehensive CRC care.

As a non-pharmacological intervention, physical exercise has garnered substantial attention for its potential to alleviate various physical and psychological side effects of cancer treatment (9). Supervised exercise programs, generally conducted in rehabilitation centers or hospitals under professional guidance, have demonstrated positive effects on physical and psychological outcomes in cancer patients (9). However, they often face barriers such as high cost, travel burden, and limited accessibility (10). In contrast, home-based exercise programs offer a promising and practical model due to their inherent advantages in accessibility, flexibility, and cost-effectiveness, making them particularly suitable for long-term, community-based cancer survivorship care (11). Specifically, within a home setting, patients can better integrate exercise into daily routines, exhibit enhanced adherence and motivation through greater autonomy in scheduling exercise times and frequencies, and maintain privacy (12). Moreover, home-based exercise is inherently more cost-effective than supervised programs in professional rehabilitation facilities, reducing both direct medical expenditures and indirect costs for patients and healthcare systems (11). It is noteworthy that a recent systematic review has highlighted the role of rehabilitation strategies, in managing constipation in oncological patients, further supporting the potential of home-based approaches across diverse cancer-related symptoms (13). These attributes make home-based exercise a patient-centered strategy for supporting long-term symptom management and improving quality of life throughout the cancer care continuum.

Empirical evidence from individual randomized controlled trials has indicated that home-based exercise programs hold potential benefits for the physical and mental health of CRC patients (14–16). However, these trials are usually limited by small sample sizes and inconsistent intervention procedures, resulting in conflicting findings regarding the effectiveness of home-based exercise on crucial outcomes such as anxiety, depression, cancer-related fatigue, and quality of life among CRC patients. To date, no meta-analysis has quantitatively synthesized the evidence from randomized controlled trials to deliver a conclusive evaluation of the effects of home-based exercise on anxiety, depression, cancer-related fatigue, and quality of life in CRC patients. In addition, although previous systematic reviews have examined exercise interventions in cancer populations, most have focused on supervised or mixed exercise settings, and none have specifically and quantitatively synthesized the evidence exclusively for home-based exercise in CRC patients across multiple psychological and physical outcomes (17, 18). Therefore, a comprehensive synthesis is urgently needed to clarify its overall efficacy, estimate the magnitude of effects, and identify the limitations in the evidence base. To address this knowledge gap, this study aimed to conduct a meta-analysis of randomized controlled trials to evaluate the effects of home-based exercise on anxiety, depression, cancer-related fatigue, and quality of life in CRC patients.

## 2 Materials and methods

This meta-analysis was registered with PROSPERO (CRD420261303142) and conducted in accordance with the Preferred Reporting Items for Systematic Reviews and Meta-Analyses (PRISMA) guidelines (19).

### 2.1 Search strategy

A comprehensive literature search was conducted in PubMed, Web of Science, Scopus, Embase, Cochrane Library, CINAHL, PsycINFO, China National Knowledge Infrastructure (CNKI), WanFang, VIP, and SinoMed (CBM). Boolean operators were utilized to synthesize a set of search terms, including MeSH terms and synonymous words, for records published from inception through February 2026. The initial search terms included “colorectal cancer,” “exercise,” “home-based exercise,” “aerobic,” “resistance training,” “physical activity,” and “randomized clinical trial.” In addition to electronic searches, the reference lists of all included studies were reviewed to identify any additional eligible publications. The search strategies are detailed in **Supplementary Table 1**.

### 2.2 Eligibility criteria

Studies were screened based on the PICOS criteria (1): Population: adult patients (≥18 years) diagnosed with CRC (2); Intervention: interventions delivered in the home setting included aerobic exercise, resistance training, physical activity programs, and progressive muscle relaxation (3); Comparison: usual care, comprising routine treatment, discharge instructions, psychiatric nursing, and exercise advice (4); Outcomes: primary outcomes were defined as anxiety, depression, cancer-related fatigue, and quality of life (5); Study design: randomized controlled trials. Exclusion criteria were (1): duplicate publications (2); conference abstracts, protocols, letters, case reports, and reviews (3); insufficient data for meta-analysis (4); not published in Chinese or English.

### 2.3 Data extraction

Data were independently extracted by two reviewers using a standardized form, with all entries subsequently verified. The extracted information encompassed first author, publication year, country, sample size, mean age, therapeutic stage, experimental group details (exercise modality, session length, frequency, and duration), and outcome measures. Any discrepancies were resolved through consultation with a third reviewer to reach a consensus.

### 2.4 Methodological quality and certainty of evidence

The methodological quality and certainty of evidence assessment were conducted independently by two reviewers, with any disagreements resolved through discussion with a third researcher. The methodological quality was assessed using the Cochrane Risk of Bias Tool 2.0 (ROB 2.0) (20). This tool evaluates the following five domains: the randomization process, deviations from the intended interventions, missing outcome data, measurement of the outcome, and selection of the reported result. Each study was then classified as having an overall “low risk,” “some concerns,” or “high risk” of bias.

The certainty of evidence regarding the effects of home-based exercise on anxiety, depression, cancer-related fatigue, and quality of life in CRC patients was evaluated using the Grading of Recommendations, Assessment, Development, and Evaluation (GRADE) framework (21). According to the GRADE framework, the certainty of evidence for each outcome was categorized into one of four levels: high, moderate, low, or very low, based on an assessment of risk of bias, inconsistency, indirectness, imprecision, and publication bias.

### 2.5 Statistical analysis

The overall effect was estimated using standardized mean differences (SMDs) with 95% confidence intervals (CIs). Statistical heterogeneity was assessed using the Q test and quantified by the I² statistic, with I² > 50% indicating substantial heterogeneity (22). Since substantial heterogeneity was detected across the included studies, a random−effects model was applied to calculate the overall effect sizes. A leave-one-out sensitivity analysis (iteratively removing one study at a time and recalculating the pooled effect) was conducted to evaluate the robustness of the results, while subgroup analyses based on therapeutic phase (active treatment vs survivors) and intervention duration (≤ 12 weeks vs > 12 weeks) were performed to explore potential sources of heterogeneity. Funnel plot and Egger’s test were employed to determine publication bias. All statistical analyses were conducted with Stata version 17.0.

## 3 Results

### 3.1 Selection results and study characteristics

**Figure 1:**
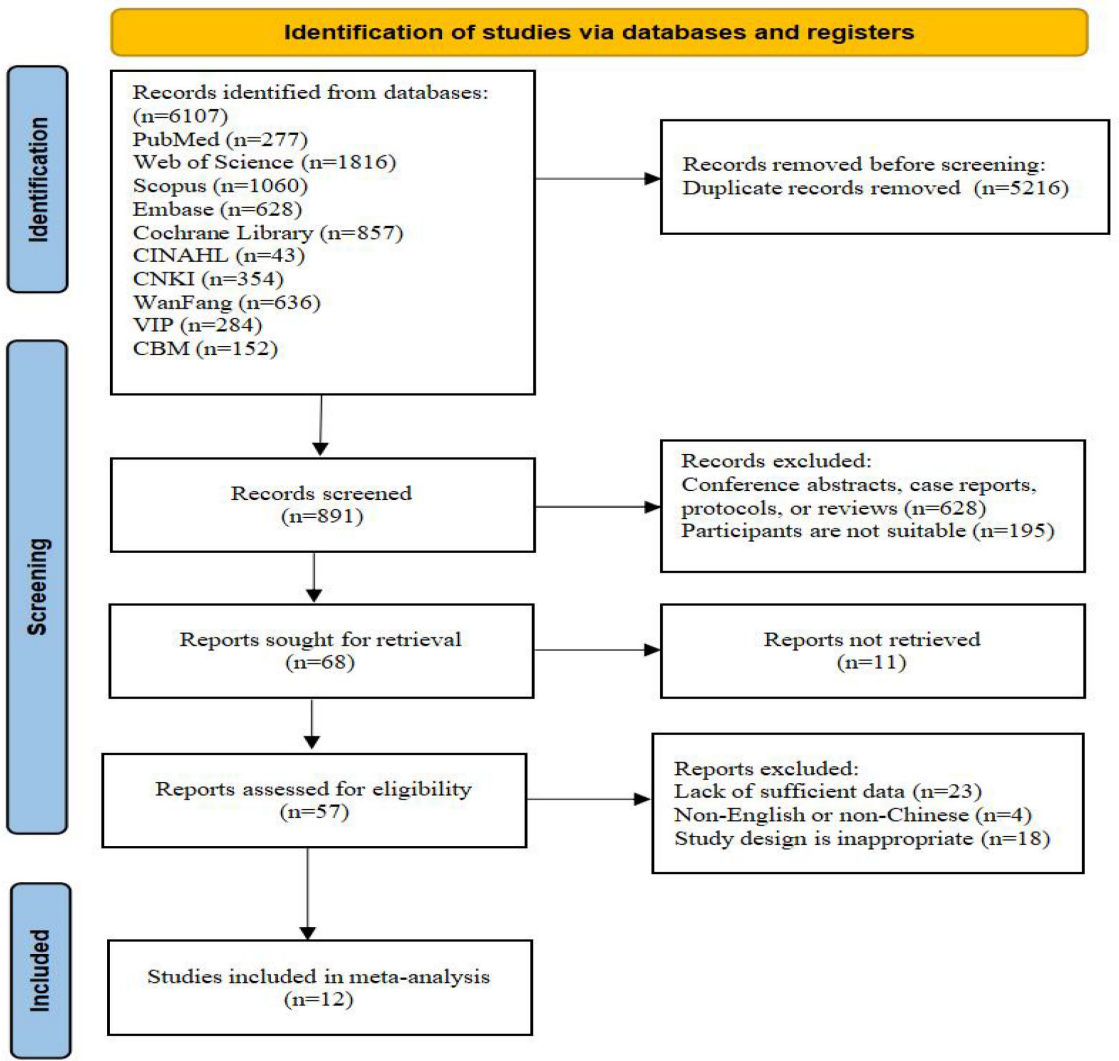
Flow diagram of study selection.

A total of 6107 records were initially identified. After removing duplicates, 891 articles were screened based on their titles and abstracts. Of these, 57 articles underwent full-text review for eligibility. Following a detailed assessment, 12 studies were finally included in the meta-analysis, as shown in **Figure 1**. The characteristics of the included studies are summarized in **Table 1**. A total of 12 studies, published between 2003 and 2025, enrolled 802 CRC patients. Most participants are receiving active treatment for CRC. Sample sizes across the individual studies ranged from 39 to 112 participants. The included trials were conducted in China (n=6), Turkey (n=2), the USA (n=2), the Netherlands (n=1), and Korea (n=1).

**Table 1:** Characteristics of included studies.

Study	Country	Participants			Experimental group				Outcomes (instrument)
		Sample size	Mean age (years)	Phase of treatment	Exercise modality	Session length	Frequency	Duration	
Brown et al., 2018 (14)	USA	EG: 26 CG: 13	NA	Survivors	Aerobic exercise	NA	NA	24 weeks	1. Cancer-related fatigue (FSI) 2. Quality of life (FACT-C)
Cheung et al., 2003 (15)	China	EG: 29 CG: 30	EG: 60.1 ± 10.91 CG: 56.4 ± 13.53	Active treatment	Progressive muscle relaxation	20 minutes	NA	10 weeks	1. Anxiety (STAI) 2. Quality of life (QOL-Colostomy)
Courneya et al., 2003 (16)	Netherlands	EG: 62 CG: 31	EG: 49.9 ± 8.4 CG: 50.5 ± 10.1	Active treatment	Aerobic exercise	20–30 minutes	3–5 sessions/week	18 weeks	1. Anxiety (STAI) 2. Depression (CES-D) 3. Cancer-related fatigue (FACT-F) 4. Quality of life (FACT-C)
Eroğlu and Kutlutürkan. 2024 (23)	Turkey	EG: 19 CG: 20	EG: 60.84 ± 11.01 CG: 60.45 ± 14.41	Active treatment	Neuromuscular control training	15 minutes	9 sessions/week	8 weeks	Quality of life (EORTC QLQ-CR29)
Ho et al., 2020 (24)	China	EG: 56 CG: 56	EG: 66.6 ± 9.5 CG: 64.9 ± 9.4	Survivors	Moderate to vigorous physical activity	30 minutes	5 sessions/week	24 weeks	1. Anxiety (HADS-A) 2. Depression (HADS-D) 3. Quality of life (FACT-C)
Kim et al., 2019 (25)	Korea	EG: 30 CG: 28	EG: 55.7 ± 8.7 CG: 56.8 ± 10.2	Survivors	Aerobic and resistance exercise	30 minutes	7 sessions/week	12 weeks	1. Depression (PHQ) 2. Cancer-related fatigue (FACT-F) 3. Quality of life (FACT-C)
Li and Gao. 2024 (26)	China	EG: 43 CG: 43	EG: 63.33 ± 6.28 CG: 63.25 ± 5.42	Active treatment	Aerobic exercise	30–45 minutes	3–4 sessions/week	8 weeks	1. Anxiety (SAS) 2. Depression (SDS) 3. Cancer-related fatigue (PFS) 4. Quality of life (EORTC QLQ-C30)
Lin et al., 2014 (27)	China	EG: 21 CG: 24	EG: 49.9 ± 8.4 CG: 50.5 ± 10.1	Active treatment	Aerobic and resistance exercise	40–60 minutes	2 sessions/week	12 weeks	1. Cancer-related fatigue (FSI) 2. Quality of life (EORTC QLQ-C30)
Ozhanli and Akyuz. 2022 (28)	Turkey	EG: 31 CG: 32	57.95 ± 11.84	Active treatment	Progressive muscle relaxation	15 minutes	1 session/day	3 days	Anxiety (STAI)
Pinto et al., 2013 (29)	USA	EG: 20 CG: 26	57.3 ± 9.7	Survivors	Aerobic exercise	10–30 minutes	7 sessions/week	12 weeks	1. Cancer-related fatigue (FACT-F) 2. Quality of life (FACT-C)
Wang and Li. 2019 (30)	China	EG: 39 CG: 39	EG: 56.77 ± 7.34 CG: 57.29 ± 6.58	Active treatment	Aerobic exercise	20–30 minutes	4 sessions/week	24 weeks	1. Cancer-related fatigue (PFS) 2. Quality of life (EORTC QLQ-C30)
Zhang and Ren. 2025 (31)	China	EG: 42 CG: 42	NA	Active treatment	Multicomponent exercise	5–20 minutes	2–3 sessions/week	12 weeks	1. Cancer-related fatigue (CFS) 3. Quality of life (SF-12)

CES-D, Center for Epidemiologic Studies Depression Scale; CFS, Cancer Fatigue Scale, EORTC QLQ-C30, European Organization for Research and Treatment of Cancer Quality of Life Questionnaire Core 30, EORTC QLQ-CR29, European Organization for Research and Treatment of Cancer Quality of Life Questionnaire-Colorectal Cancer, FACT-C, Functional Assessment of Cancer Therapy-Colorectal, FACT-F, Functional Assessment of Cancer Therapy-Fatigue; FSI, Fatigue Symptom Inventory, HADS-A, Hospital Anxiety and Depression Scale-Anxiety, HADS-D, Hospital Anxiety and Depression Scale-Depression; PFS, Piper Fatigue Scale; PHQ, Patient Health Questionnaire, QOL-Colostomy, Quality of Life Index for Colostomy; SAS, Self-Rating Anxiety Scale; SDS, Self-Rating Depression Scale, SF-12 = 12-Item Short Form Health Survey; STAI, State-Trait Anxiety Inventory.

### 3.2 Summary of risk of bias

**Figure 2:**
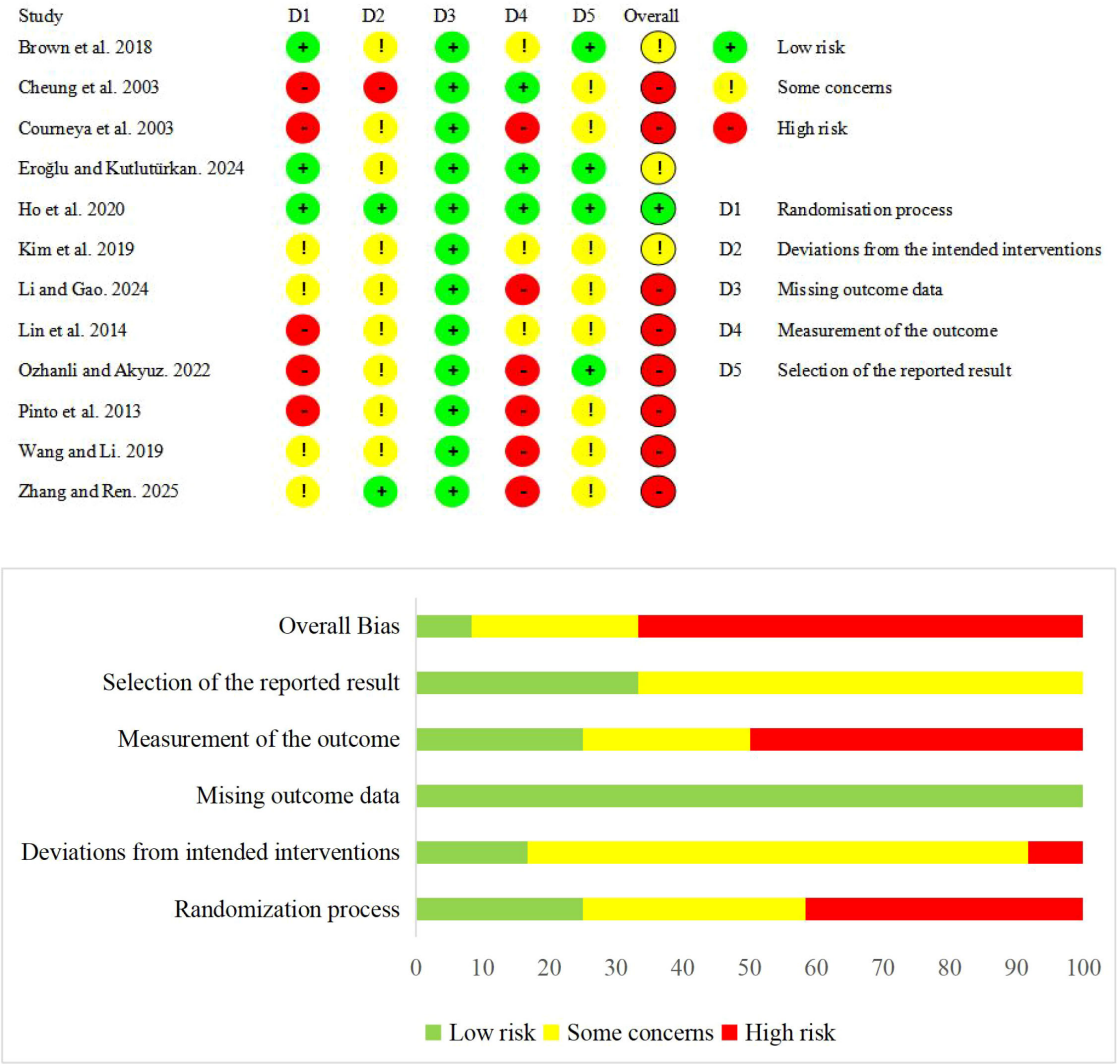
Summary of risk of bias.

The methodological quality of the included studies is summarized in **Figure 2**. Overall, 8 studies were rated as high risk of bias, 3 as having some concerns, and 1 as low risk. Across specific bias domains, the randomization process was judged as low risk in 3 studies, some concerns in 4, and high risk in 5. Deviations from intended interventions were considered low risk in 2 studies, raised some concerns in 9, and high risk in 1. Although missing outcome data was consistently low risk across all studies, outcome measurement was rated as low risk in 3 studies, some concerns in 3, and high risk in 6. Regarding the selection of reported results, 4 studies were at low risk, and 8 raised some concerns.

### 3.3 Main outcomes of the meta-analysis

#### 3.3.1 Anxiety

**Figure 3:**
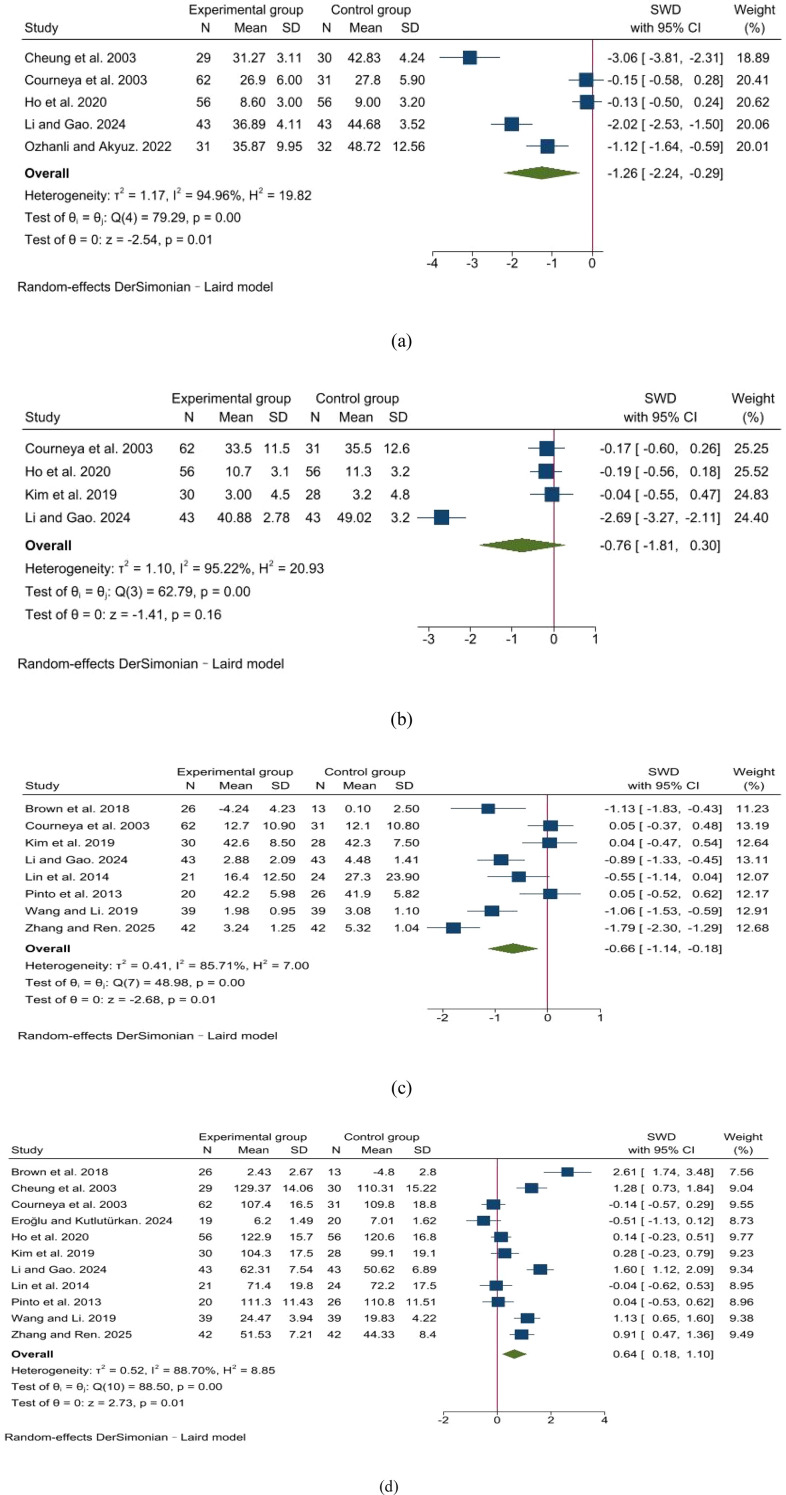
The effects of home-based exercise on anxiety, depression, cancer-related fatigue, and quality of life in CRC patients. **(a)** anxiety; **(b)** depression; **(c)** cancer-related fatigue; **(d)** quality of life.

As shown in **Figure 3A**, the pooled analysis of 5 trials demonstrated that home-based exercise significantly reduced anxiety in CRC patients compared to usual care (SMD = -1.26, 95% CI: -2.24 to -0.29), with a high heterogeneity (I² = 95%). Stratified analysis based on therapeutic phase revealed that patients undergoing active treatment experienced a significant reduction in anxiety after performing home-based exercises (SMD = -1.56, 95% CI: -2.73 to -0.40), whereas no improvement was observed among survivors (SMD = -0.13, 95% CI: -0.50 to -0.24) (**Supplementary Figure 1A**). Furthermore, subgroup analysis indicated that home-based exercise with a duration of ≤12 weeks significantly alleviated anxiety in CRC patients (SMD = -2.04, 95% CI: -3.05 to -1.02), with a more pronounced effect compared to interventions lasting >12 weeks (SMD = -0.14, 95% CI: -0.42 to -0.14) (**Supplementary Figure 1B**).

#### 3.3.2 Depression

5 trials investigated the effect of home-based exercise in alleviating depression in CRC patients. The pooled analysis (**Figure 3B**) showed that, compared to usual care, home-based exercise was not associated with a statistically significant reduction in anxiety among CRC patients (SMD = -0.76, 95% CI: -1.81 to 0.30), despite substantial heterogeneity (I² = 95%). Subgroup analyses revealed no significant differences in the alleviation of depression in CRC patients based on therapeutic phase or intervention duration (**Supplementary Figure 2**).

#### 3.3.3 Cancer-related fatigue

A meta-analysis of 8 trials assessing cancer-related fatigue revealed that home-based exercise significantly reduced fatigue in CRC patients compared to usual care (SMD = -0.66, 95% CI: -1.14 to -0.18, I² = 86%) (**Figure 3C**). Subgroup analyses identified no significant association between variations in therapeutic phase or intervention duration of home-based exercise and the effect of alleviating cancer-related fatigue in CRC patients (**Supplementary Figure 3**).

#### 3.3.4 Quality of life

A meta-analysis of the 11 trials assessing quality of life showed that home-based exercise was associated with a significant improvement compared to usual care in CRC patients (SMD = 0.64, 95% CI: 0.18 to 1.10; I² = 89%; **Figure 3D**). Stratified analyses indicated that neither therapeutic phase nor intervention duration significantly moderated the effectiveness of home-based exercise on quality of life in CRC patients (**Supplementary Figure 4**).

### 3.4 Sensitivity analyses

The results of the sensitivity analysis are detailed in **Supplementary Figure 5**. Specifically, sensitivity analyses for anxiety, cancer-related fatigue, and quality of life demonstrated the robustness of the pooled results. In contrast, sensitivity analysis for depression indicated that the pooled estimate was unstable.

### 3.5 Publication bias assessment

Given that more than 10 studies reported on quality of life, publication bias was assessed using a funnel plot and Egger’s test. The results of both the funnel plot (**Supplementary Figure 6**) and Egger’s test (**Supplementary Figure 7**) indicated no significant publication bias.

### 3.6 The certainty of Evidence

According to the GRADE framework, the certainty of evidence was judged as low for the effectiveness of home-based exercise in reducing anxiety and cancer-related fatigue, as well as improving quality of life in CRC patients, while it was assessed as very low for its effect on depression. Details of these ratings and the associated rationale are provided in **Supplementary Table 2**.

## 4 Discussion

This study synthesized data from 12 randomized controlled trials investigating the effects of home-based exercise on anxiety, depression, cancer-related fatigue, and quality of life in CRC patients. Our findings revealed that home-based exercise demonstrated positive effects in mitigating anxiety and cancer-related fatigue, as well as enhancing quality of life among CRC patients. In contrast, no significant reduction in their depression was observed. The overall certainty of evidence for the combined outcomes was rated as low to very low, primarily due to the high risk of bias in the included studies and substantial heterogeneity across findings. Although the observed heterogeneity could not be sufficiently explained by subgroup analyses based on therapeutic phase or intervention duration, it may be attributable to variations in sample characteristics, intervention formats, and outcome measures among the included studies. Given the low to very low certainty of evidence, small total sample size (802 participants across 12 studies), and substantial heterogeneity, these findings should be interpreted as hypothesis-generating rather than conclusive. Therefore, further high-quality randomized controlled trials are warranted to draw more definitive conclusions.

CRC patients generally experience negative emotions such as anxiety and depression, which impair their quality of life (4, 5). Our findings indicated that home-based exercise significantly reduced anxiety in CRC patients without alleviating depression. However, the wide confidence interval indicates imprecision, and this large effect should be interpreted with caution. Regular exercise has been confirmed to reduce levels of stress hormones such as cortisol while promoting the release of endorphins, directly improving emotional well-being (17). Furthermore, structured home-based exercise programs provide CRC patients with constructive physical activity, diverting their attention away from disease-related concerns and physical discomfort (32). This contributes to the cultivation of a more positive body image and more optimistic expectations regarding recovery, thereby alleviating anxiety and feelings of helplessness. It is noteworthy that subgroup analysis revealed that home-based exercise lasting ≤ 12 weeks significantly reduced anxiety in CRC patients more effectively than exercise lasting > 12 weeks. This may be attributable to higher adherence rates in shorter, more focused programs, whereas longer interventions might suffer from declining participant engagement over time. From a clinical perspective, the reduction in anxiety corresponds to a large effect size. For CRC patients, this magnitude of improvement may translate into less distress during oncology follow-up, better treatment adherence, and improved daily functioning (25). Depression in cancer patients is a multifaceted construct, intertwined with deep-seated alterations (5). The relatively brief and standardized nature of home-based exercise may be insufficient to address the core pathological mechanisms of clinical depression. Furthermore, the variability in assessment tools across studies could have diluted the ability to detect a consistent signal of improvement. Sensitivity analysis showed that the pooled results of the effect of home-based exercise on depression were unstable, and the certainty of evidence was rated as very low. Additionally, the small total sample size and the generally mild baseline depression levels in most included studies may have limited the ability to detect a significant effect. Therefore, future well-designed randomized controlled trials using standardized and validated depression assessment tools are needed to clarify the effect of home-based exercise on depression in CRC patients.

Cancer-related fatigue exerts a multidimensional burden on CRC patients (6). CRC patients frequently engage in less activity to conserve energy to cope with fatigue, which leads to a decline in physical fitness and, in turn, increased susceptibility to fatigue (33). Our study indicated that home-based exercise could significantly reduce cancer-related fatigue in CRC patients. The efficacy of home-based exercise in alleviating cancer-related fatigue in CRC patients could be explained by interrelated physiological and psychological mechanisms. From a physiological perspective, home-based exercise enhances muscle strength, improves cardiopulmonary function, and boosts energy metabolism efficiency (34). This accelerates the clearance of inflammatory factors, boosts neural excitability, and reduces physical fatigue. Moreover, during physical activity, patients’ attention gradually shifts away from feelings of fatigue and discomfort, alleviating anxiety and depressive moods (35). Consequently, the subjective perception of fatigue is significantly diminished.

Enhancing the quality of life in CRC patients is a central objective in oncology care (36). As a multidimensional outcome, quality of life in CRC patients involves critical domains such as physical, role, emotional, social, and cognitive functioning (37). This meta-analysis demonstrated that home-based exercise leads to a significant improvement in quality of life among CRC patients. Firstly, home-based exercise encompasses various types such as aerobic exercise, resistance training, or multi-component exercise. These activities enhance muscle strength and physical function, enabling patients to better engage in daily living activities, reduce dependence on others, and regain physical independence (38). Secondly, exercise effectively alleviates anxiety and depression symptoms by providing distraction, thereby improving emotional functioning (32). Thirdly, exercise promotes the restoration of physical stamina and energy, empowering patients to re-engage in family activities, social interactions, and leisure pursuits (39). Finally, regular aerobic exercise increases cerebral blood flow, ensuring neurons receive adequate oxygen and energy substrates. Meanwhile, it enhances cerebral insulin sensitivity and energy metabolism efficiency, optimizing the functional environment for brain cells and thereby building stronger cognitive reserves (40).

Some limitations should be considered. First, this study exclusively included Chinese and English literature, which may introduce potential publication bias. Future systematic reviews should consider including multiple language databases to minimize such bias. Second, the sample size employed for the meta-analysis is relatively small, which may result in insufficient statistical power and external validity of the findings. To address this, future randomized controlled trials with larger sample sizes and adequate power calculations are needed. Additionally, trial sequential analysis could be applied in future updates to confirm whether the evidence is sufficient or more trials are required. Finally, all outcome measures were assessed using self-report scales, which may introduce recall bias. Future research should employ objective measures or multi-informant approaches and incorporate validated, multidimensional outcome measures to improve the accuracy and robustness of the findings.

### 4.1 Implications for clinical practice

The results of this meta-analysis have several practical implications for clinical practice and survivorship care in CRC patients. First, home-based exercise is highly feasible for CRC patients who have completed active treatment and can perform unsupervised physical activity. It removes common barriers associated with supervised programs, such as travel distance and facility costs. Second, given that adherence is a critical determinant of effectiveness, strategies such as wearable activity trackers, smartphone reminders, weekly phone coaching, or exercise diaries could enhance long-term compliance. Our subgroup analysis suggesting that shorter interventions (≤ 12 weeks) yield better anxiety reduction also implies that initial shorter programs with gradual extension may optimize adherence. Third, patients with mild to moderate anxiety or fatigue, adequate self-efficacy, and a safe home environment are most likely to benefit. Those with severe depression or significant functional limitations may require initial supervised support before transitioning to home-based exercise. Therefore, healthcare providers should consider prescribing a tailored home-based exercise plan as part of a comprehensive cancer rehabilitation pathway, taking into account individual preferences, clinical status, and available resources (14).

## 5 Conclusions

Considering the low heterogeneity and low certainty of the evidence, this meta-analysis suggests that home-based exercise may be effective in reducing anxiety and cancer-related fatigue, as well as improving quality of life in CRC patients. In contrast, no statistically significant effect was observed on depression levels in this population. Therefore, further well-designed randomized controlled trials are essential to establish a more robust evidence base.

## References

[1] Gao L Feng L Wang C Zhao X Xiong M Huang Q . The relationship between the latent profiles of cancer-related fatigue characteristics and exercise adherence in colorectal cancer patients. Support Care Cancer. (2026) 34:172. doi: 10.1007/s00520-026-10399-2. PMID: 41652085 PMC12881022

[2] Morgan E Arnold M Gini A Lorenzoni V Cabasag CJ Laversanne M . Global burden of colorectal cancer in 2020 and 2040: incidence and mortality estimates from GLOBOCAN. Gut. (2023) 72:338–44. doi: 10.1136/gutjnl-2022-327736. PMID: 36604116

[3] Ziętarska M Małgorzewicz S . Quality of life of colorectal cancer patients treated with chemotherapy. Nutrients. (2026) 18:191. doi: 10.3390/nu18020191. PMID: 41599804 PMC12845471

[4] Yuan P Wang D Xie D . Anxiety and depression after colorectal cancer surgery: a systematic review and meta-analysis of short- and long-term outcomes. Alpha Psychiatry. (2024) 25:429–39. doi: 10.5152/alphapsychiatry.2024.231359. PMID: 39360300 PMC11443286

[5] Makrygianni P Polikandrioti M Koutelekos I Tsiampouris I Vasilopoulos G . Anxiety and depression in patients with colorectal cancer undergoing ileostomy: a systematic review. Adv Exp Med Biol. (2026) 1487:493–501. doi: 10.1007/978-3-032-03398-7_44. PMID: 41273586

[6] Romero-Elías M Álvarez-Bustos A Méndez M Sánchez A Gutiérrez L Cebolla-Boado H . Correlates of cancer-related fatigue in colorectal cancer patients at the time of diagnosis. Support Care Cancer. (2025) 33:338. doi: 10.1007/s00520-025-09386-w. PMID: 40167768

[7] Brown KA Cordts KP Lally RM . Quality of life and unmet needs of late-stage and metastatic colorectal cancer survivors: an integrative review. J Psychosoc Oncol. (2025) 43:616–31. doi: 10.1080/07347332.2024.2425679. PMID: 39523831

[8] Orive M Anton-Ladislao A Lázaro S Gonzalez N Bare M Fernandez de Larrea N . Anxiety, depression, health-related quality of life, and mortality among colorectal patients: 5-year follow-up. Support Care Cancer. (2022) 30:7943–54. doi: 10.1007/s00520-022-07177-1. PMID: 35737143 PMC9512719

[9] Granger CL Edbrooke L Antippa P Wright G McDonald CF Zannino D . Home-based exercise and self-management after lung cancer resection: a randomized clinical trial. JAMA Netw Open. (2024) 7:e2447325. doi: 10.1001/jamanetworkopen.2024.47325. PMID: 39621348 PMC11612835

[10] Chen S Lv C Wu J Zhou C Shui X Wang Y . Effectiveness of a home-based exercise program among patients with lower limb spasticity post-stroke: a randomized controlled trial. Asian Nurs Res (Korean Soc Nurs Sci). (2021) 15:1–7. doi: 10.1016/j.anr.2020.08.007. PMID: 32890770

[11] de Menezes KKP Ada L Teixeira-Salmela LF Scianni AA Avelino PR Faria CDCM . Home-based interventions may increase recruitment, adherence, and measurement of outcomes in clinical trials of stroke rehabilitation. J Stroke Cerebrovasc Dis. (2021) 30:106022. doi: 10.1016/j.jstrokecerebrovasdis.2021.106022. PMID: 34364011

[12] Mahmood A Deshmukh A Natarajan M Marsden D Vyslysel G Padickaparambil S . Development of strategies to support home-based exercise adherence after stroke: a Delphi consensus. BMJ Open. (2022) 12:e055946. doi: 10.1136/bmjopen-2021-055946. PMID: 34992120 PMC8739434

[13] Chiaramonte R Bonfiglio M Caramma S Condorelli R . The role of rehabilitation in the treatment of constipation in oncological patients. J Clin Med. (2023) 12:5083. doi: 10.3390/jcm12155083. PMID: 37568485 PMC10420032

[14] Brown JC Damjanov N Courneya KS Troxel AB Zemel BS Rickels MR . A randomized dose-response trial of aerobic exercise and health-related quality of life in colon cancer survivors. Psychooncology. (2018) 27:1221–8. doi: 10.1002/pon.4655. PMID: 29388275 PMC5895514

[15] Cheung YL Molassiotis A Chang AM . The effect of progressive muscle relaxation training on anxiety and quality of life after stoma surgery in colorectal cancer patients. Psychooncology. (2003) 12:254–66. doi: 10.1002/pon.638. PMID: 12673809

[16] Courneya KS Friedenreich CM Quinney HA Fields AL Jones LW Fairey AS . A randomized trial of exercise and quality of life in colorectal cancer survivors. Eur J Cancer Care (Engl). (2003) 12:347–57. doi: 10.1046/j.1365-2354.2003.00437.x. PMID: 14982314

[17] Soong RY Low CE Ong V Sim I Lee C Lee F . Exercise interventions for depression, anxiety, and quality of life in older adults with cancer: a systematic review and meta-analysis. JAMA Netw Open. (2025) 8:e2457859. doi: 10.1001/jamanetworkopen.2024.57859. PMID: 39903465 PMC11795328

[18] Michael CM Lehrer EJ Schmitz KH Zaorsky NG . Prehabilitation exercise therapy for cancer: a systematic review and meta-analysis. Cancer Med. (2021) 10:4195–205. doi: 10.1002/cam4.4021. PMID: 34110101 PMC8267161

[19] Brennan SE Munn Z . PRISMA 2020: a reporting guideline for the next generation of systematic reviews. JBI Evid Synth. (2021) 19:906–8. doi: 10.11124/JBIES-21-00112. PMID: 33989266

[20] Sterne JAC Savović J Page MJ Elbers RG Blencowe NS Boutron I . RoB 2: a revised tool for assessing risk of bias in randomized trials. BMJ. (2019) 366:l4898. doi: 10.1136/bmj.l4898. PMID: 31462531

[21] Guyatt G Oxman AD Akl EA Kunz R Vist G Brozek J . GRADE guidelines: 1. Introduction-GRADE evidence profiles and summary of findings tables. J Clin Epidemiol. (2011) 64:383–94. doi: 10.1016/j.jclinepi.2010.04.026. PMID: 21195583

[22] White IR Thomas J . Standardized mean differences in individually-randomized and cluster-randomized trials, with applications to meta-analysis. Clin Trials. (2005) 2:141–51. doi: 10.1191/1740774505cn081oa. PMID: 16279136

[23] Eroğlu İ Kutlutürkan S . The effect of hand-foot exercises on chemotherapy-induced peripheral neuropathy-related pain, falls, and quality of life in colorectal cancer: a randomized controlled trial. Eur J Oncol Nurs. (2024) 71:102641. doi: 10.1016/j.ejon.2024.102641. PMID: 38897103

[24] Ho M Ho JWC Fong DYT Lee CF Macfarlane DJ Cerin E . Effects of dietary and physical activity interventions on generic and cancer-specific health-related quality of life, anxiety, and depression in colorectal cancer survivors: a randomized controlled trial. J Cancer Surviv. (2020) 14:424–33. doi: 10.1007/s11764-020-00864-0. PMID: 32072434 PMC7360640

[25] Kim JY Lee MK Lee DH Kang DW Min JH Lee JW . Effects of a 12-week home-based exercise program on quality of life, psychological health, and the level of physical activity in colorectal cancer survivors: a randomized controlled trial. Support Care Cancer. (2019) 27:2933–40. doi: 10.1007/s00520-018-4588-0. PMID: 30564936

[26] Li NN Gao XY . Effects of aerobic exercise nursing in patients undergoing postoperative chemotherapy for colorectal cancer. In: Chinese journal of medicated people, vol. 36. (2024). p. 177–9. Beijing, China: Chinese Journal of Medicated People Publishing House.

[27] Lin KY Shun SC Lai YH Liang JT Tsauo JY . Comparison of the effects of a supervised exercise program and usual care in patients with colorectal cancer undergoing chemotherapy. Cancer Nurs. (2014) 37:E21–9. doi: 10.1097/NCC.0b013e3182791097. PMID: 23357886

[28] Ozhanli Y Akyuz N . The effect of progressive relaxation exercise on physiological parameters, pain and anxiety levels of patients undergoing colorectal cancer surgery: a randomized controlled study. J Perianesth Nurs. (2022) 37:238–46. doi: 10.1016/j.jopan.2021.08.008. PMID: 34903440

[29] Pinto BM Papandonatos GD Goldstein MG Marcus BH Farrell N . Home-based physical activity intervention for colorectal cancer survivors. Psychooncology. (2013) 22:54–64. doi: 10.1002/pon.2047. PMID: 21905158

[30] Wang L Li XL . Observation on the application effect of aerobic exercise in patients undergoing adjuvant chemotherapy after colorectal cancer surgery. In: Modern digestion & Intervention, vol. 24. (2019). p. 1149–52. Guangzhou, China: Editorial Office of Modern Digestion & Intervention.

[31] Zhang QX Ren GP . Application of home-based multicomponent exercise nursing intervention program in elderly patients with colorectal cancer. In: Chinese general practice nursing, vol. 23. (2025). p. 1344–7. Taiyuan, China: Shanxi Medical Periodical Press Co., Ltd.

[32] Loh KP Kleckner IR Lin PJ Mohile SG Canin BE Flannery MA . Effects of a home-based exercise program on anxiety and mood disturbances in older adults with cancer receiving chemotherapy. J Am Geriatr Soc. (2019) 67:1005–11. doi: 10.1111/jgs.15951. PMID: 31034591 PMC6544022

[33] Wang S Song Y Zhang H Song J Guo X Jiang X . Cancer-related fatigue and its influencing factors among colorectal cancer patients: a generalized linear modeling approach. Int J Gen Med. (2024) 17:579–95. doi: 10.2147/IJGM.S447697. PMID: 38374816 PMC10876184

[34] Houben LHP Overkamp M Van Kraaij P Trommelen J Roermund VAN JGH Vries DE P . Resistance exercise training increases muscle mass and strength in prostate cancer patients on androgen deprivation therapy. Med Sci Sports Exerc. (2023) 55:614–24. doi: 10.1249/MSS.0000000000003095. PMID: 36534950 PMC9997646

[35] Banyard H Edward KL Garvey L Stephenson J Azevedo L Benson AC . The effects of aerobic and resistance exercise on depression and anxiety: systematic review with meta-analysis. Int J Ment Health Nurs. (2025) 34:e70054. doi: 10.1111/inm.70054. PMID: 40432290 PMC12117297

[36] Antoniadis D Giakoustidis A Papadopoulos V Fountoulakis KN Watson M . Quality of life, distress and psychological adjustment in patients with colon cancer. Eur J Oncol Nurs. (2024) 68:102467. doi: 10.1016/j.ejon.2023.102467. PMID: 38006715

[37] Flórez JES Zapata JL Duarte MCP Acevedo VV Calle JAZ Montoya AR . Factors associated with quality of life among colorectal cancer patients: cross-sectional study. Cancer Control. (2024) 31:10732748241302915. doi: 10.1177/10732748241302915. PMID: 39557561 PMC11574891

[38] Chen R Guo Y Kuang Y Zhang Q . Effects of home-based exercise interventions on post-stroke depression: a systematic review and network meta-analysis. Int J Nurs Stud. (2024) 152:104698. doi: 10.1016/j.ijnurstu.2024.104698. PMID: 38290424

[39] Yang L Yuan Z Peng C . Effects of aerobic exercise on cognitive function and quality of life in patients with Alzheimer's disease: a systematic review and meta-analysis. BMJ Open. (2025) 15:e090623. doi: 10.1136/bmjopen-2024-090623. PMID: 39800395 PMC11752035

[40] Lenze EJ Voegtle M Miller JP Ances BM Balota DA Barch D . Effects of mindfulness training and exercise on cognitive function in older adults: a randomized clinical trial. JAMA. (2022) 328:2218–29. doi: 10.1001/jama.2022.21680. PMID: 36511926 PMC9856438

