# Supplementary material for: Effects of home-based exercise on anxiety, depression, cancer-related fatigue, and quality of life in colorectal cancer patients: a meta-analysis of randomized controlled trials
Source: Front Oncol. 2026 May 7;16:1810117. doi: 10.3389/fonc.2026.1810117 (PMC13189742; doi:10.3389/fonc.2026.1810117)
Supplement: Supplementary file 1 [file DataSheet1.docx]

**Supplemental Materials**

Table S1. The search strategies.

| Databases | Step | Search Strategies |
| --- | --- | --- |
| PubMed | #1 | "colorectal [neoplasms](https://www.ncbi.nlm.nih.gov/mesh/68009369)"[Mesh Terms] Sort by: Best match |
|  | #2 | "colorectal cancer"[Title/Abstract] OR "colon cancer"[Title/Abstract] OR "rectal cancer"[Title/Abstract] |
|  | #3 | "exercise"[Mesh Terms] Sort by: Best match |
|  | #4 | "exercise"[Title/Abstract] OR "home-based exercise"[Title/Abstract] OR "aerobic"[Title/Abstract] OR "resistance training"[Title/Abstract] OR "physical activity"[Title/Abstract] Sort by: Best match |
|  | #5 | "RCT"[Title/Abstract] OR "randomized clinical trial"[Title/Abstract] OR "randomized controlled trial"[Title/Abstract] OR "randomized trial"[Title/Abstract] OR "randomised controlled trial"[Title/Abstract] OR "randomised trial"[Title/Abstract] Sort by: Most Recent |
|  | #6 | #1 OR #2 Sort by: Best match |
|  | #7 | #3 OR #4 Sort by: Best match |
|  | #8 | #5 AND #6 AND #7 Sort by: Best match |
| Web of Science | #1 | **TS=(**colorectal [neoplasms](https://www.ncbi.nlm.nih.gov/mesh/68009369)**) OR TS=(**colorectal **cancer**) OR TS=(colon cancer) OR TS=(rectal cancer) |
|  | #2 | **Ts=(exercise) OR Ts=(home-based exercise) OR** TS=(aerobic) **OR** TS=(resistance training) **OR** TS=(physical activity) |
|  | #3 | TS=(RCT) OR **TS=(**randomized clinical trial**) OR TS=(**randomized controlled trial) OR TS=(randomized trial) OR TS=(randomised controlled trial) OR TS=(randomised trial) |
|  | #4 | #1 AND #2 AND #3 |
| Scopus | #1 | ABS("colorectal [neoplasms](https://www.ncbi.nlm.nih.gov/mesh/68009369)" OR "colorectal cancer" OR "colon cancer" OR "rectal cancer") |
|  | #2 | ABS("exercise" OR "home-bases exercise" OR "aerobic" OR "resistance training" OR "physical activity") |
|  | #3 | ABS ("RCT" OR "randomized clinical trial" OR "randomized controlled trial" OR "randomized trial" OR "randomised controlled trial OR "randomised trial) |
|  | #4 | #1 AND #2 AND #3 |
| Embase | **#1** | **'**colorectal cancer**'/exp** |
|  | **#2** | 'colorectal cancer':ti,ab,kw OR 'colon cancer':ti,ab,kw OR 'rectal cancer':ti,ab,kw |
|  | **#3** | **'exercise'/exp** |
|  | **#4** | 'exercise**':ti,ab,kw OR '**home-bases exercise**':ti,ab,kw OR '**aerobic**':ti,ab,kw OR '**resistance training**':ti,ab,kw OR '**physical activity**':ti,ab,kw** |
|  | **#5** | 'RCT**':ti,ab,kw OR '**randomized clinical trial**':ti,ab,kw OR '**randomized controlled trial**':ti,ab,kw OR '**randomized trial**':ti,ab,kw OR '**randomised controlled trial**':ti,ab,kw OR '**randomised trial**':ti,ab,kw** |
|  | #6 | #1 OR #2 |
|  | #7 | #3 OR #4 |
|  | #8 | #5 AND #6 AND #7 |
| Cochrane Library | #1 | MeSH descriptor: [Colorectal Neoplasms] explode all trees |
|  | #2 | (colorectal cancer):ti,ab,kw OR (colon cancer):ti,ab,kw OR (rectal cancer):ti,ab,kw |
|  | #3 | MeSH descriptor: [Exercise] explode all trees |
|  | #4 | (exercise)**:ti,ab,kw OR (home-based exercise**)**:ti,ab,kw OR (**aerobic)**:ti,ab,kw OR (**resistance training)**:ti,ab,kw OR (**physical activity)**:ti,ab,kw** |
|  | #5 | (RCT)**:ti,ab,kw OR (**randomized clinical trial)**:ti,ab,kw OR (**randomized controlled trial)**:ti,ab,kw OR (**randomized trial)**:ti,ab,kw OR (**randomised controlled trial)**:ti,ab,kw OR (**randomised trial)**:ti,ab,kw** |
|  | #6 | #1 OR #2 |
|  | #7 | #3 OR #4 |
|  | #8 | #5 AND #6 AND #7 |
| CINAHL | S1 | **TI** colorectal [neoplasms](https://www.ncbi.nlm.nih.gov/mesh/68009369) **OR TI** colorectal **cancer** OR TI colon cancer OR TI rectal cancer |
|  | S2 | **TI exercise OR TI home-based exercise OR** TI aerobic **OR** TI resistance training **OR** TI physical activity |
|  | S3 | TI RCT **OR TI** randomized clinical trial **OR TI** randomized controlled trial **OR TI** randomized trial **OR TI** randomised controlled trial **OR TI** randomised trial |
|  | S4 | #1 AND #2 AND #3 |
| CNKI |  | (SU="结直肠癌"+ "结肠癌" + "直肠癌") AND (SU="运动" + "居家运动"+ "居家锻炼"+ "有氧运动"+ "抗阻运动") |
| WanFang |  | 题名或关键词:(结直肠癌 OR 结肠癌 OR 直肠癌) and 题名或关键词:(运动 OR 居家运动 OR 居家锻炼 OR 有氧运动OR 抗阻运动) |
| VIP |  | (题名或关键词=结直肠癌 OR 结肠癌 OR 直肠癌) and (题名或关键词=运动 OR 居家运动 OR 居家锻炼OR 有氧运动OR 抗阻运动) |
| CBM |  | ("结直肠癌"[标题] OR "结肠癌"[标题] OR "直肠癌"[标题] ) AND ( "运动"[标题] OR "居家运动"[标题] OR "居家锻炼"[标题] OR "有氧运动"[标题] OR "抗阻运动"[标题]) |


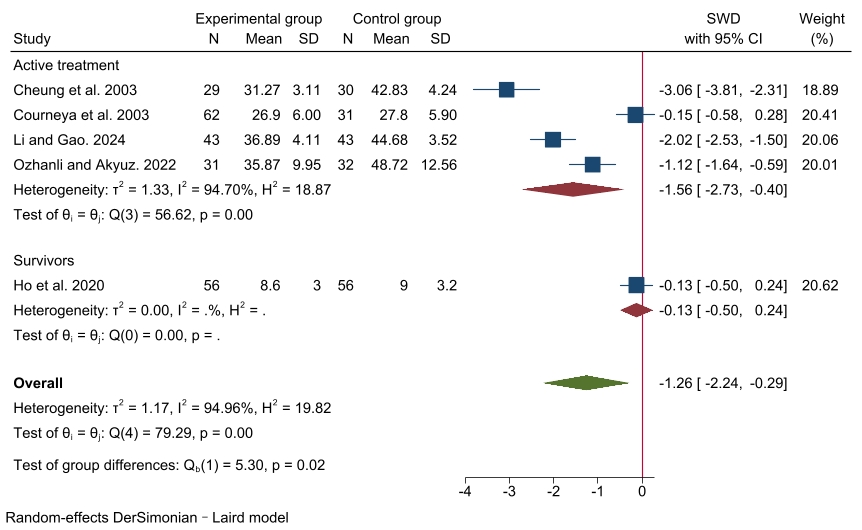


(a)


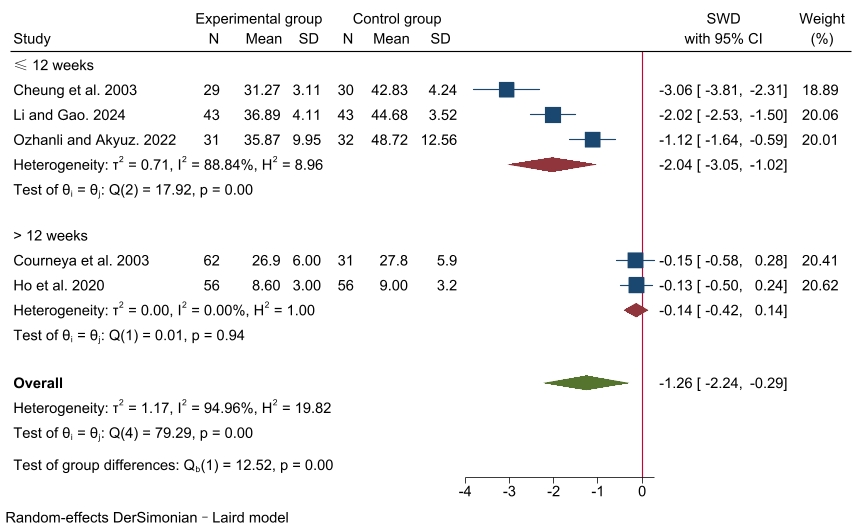


(b)

Figure S1. The subgroup analysis for anxiety. (a) Based on therapeutic phase; (b) Based on intervention duration.

**
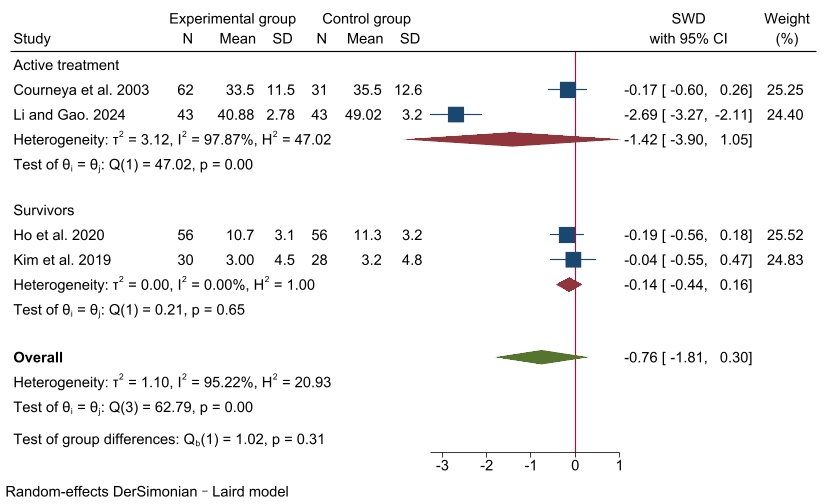
**

(a)


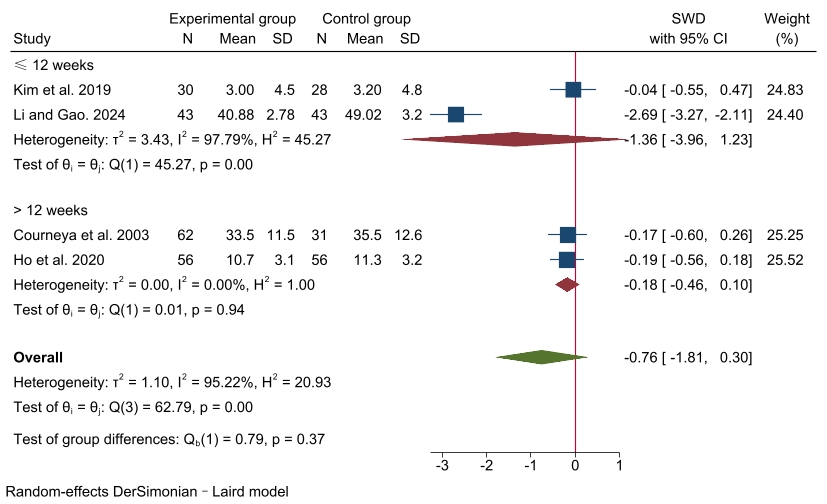
(b)

Figure S2. The subgroup analysis for depression. (a) Based on therapeutic phase; (b) Based on intervention duration.


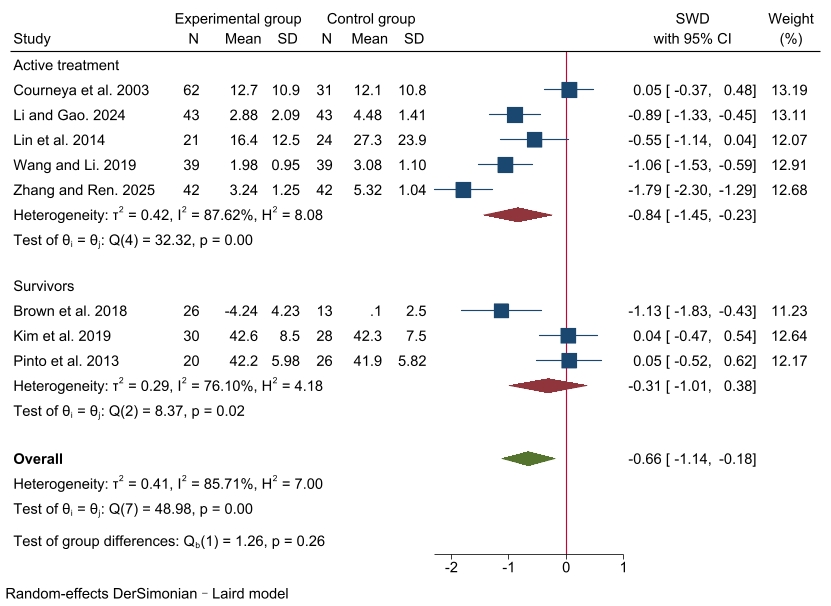
(a)


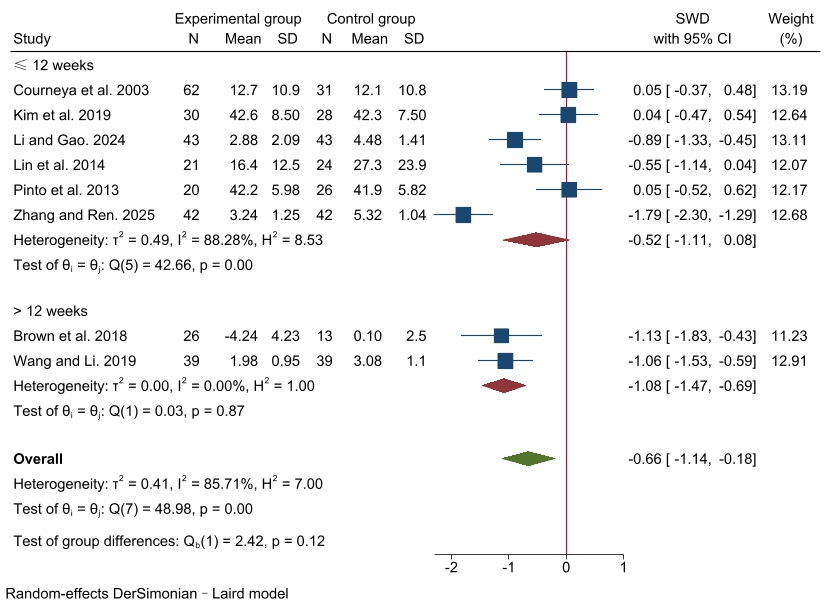
(b)

Figure S3. The subgroup analysis for cancer-related fatigue. (a) Based on therapeutic phase; (b) Based on intervention duration.


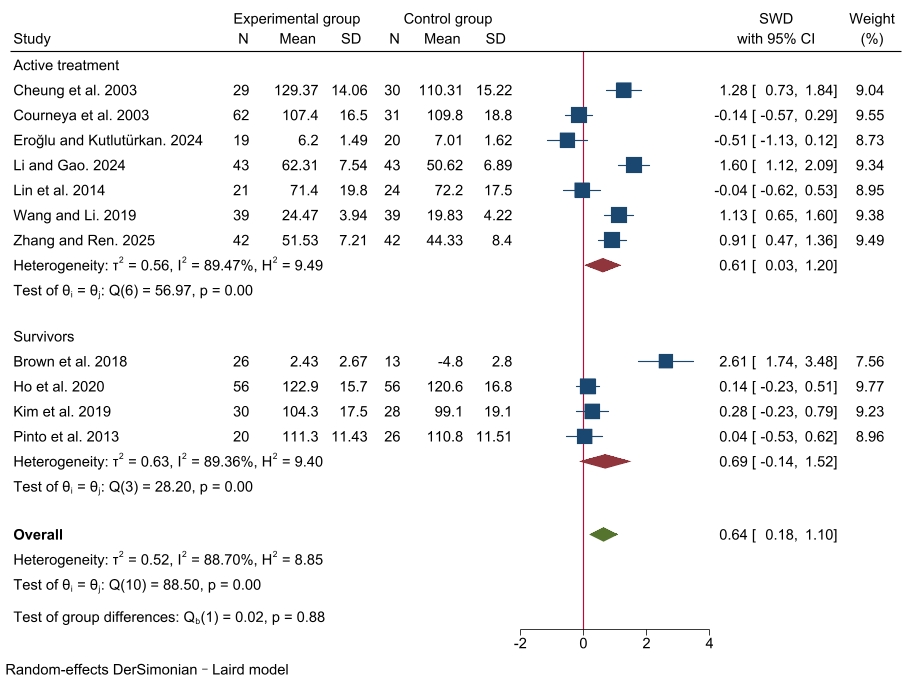
(a)


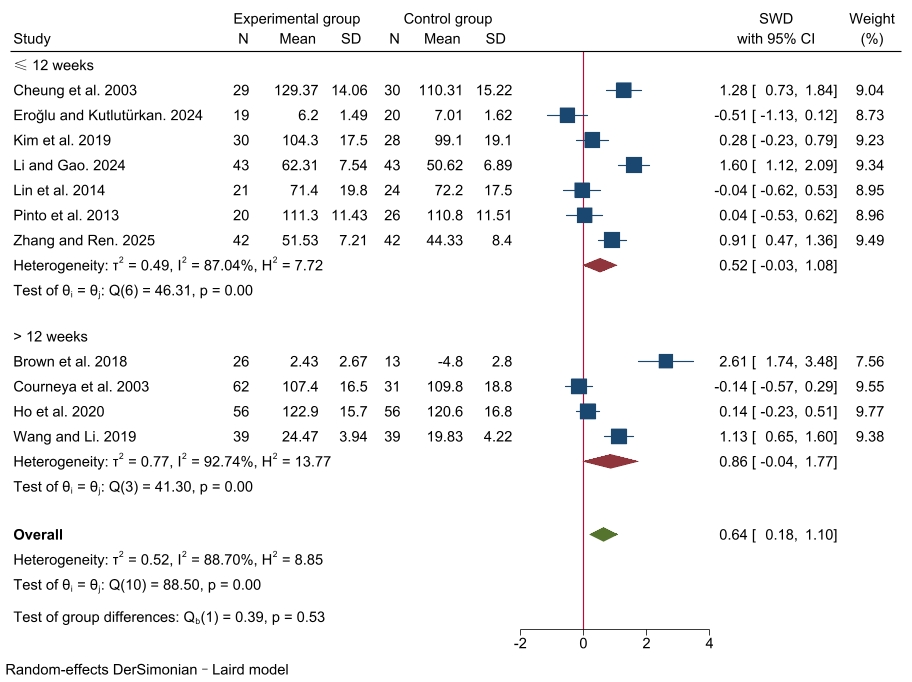
(b)

Figure S4. The subgroup analysis for quality of life. (a) Based on therapeutic phase; (b) Based on intervention duration.


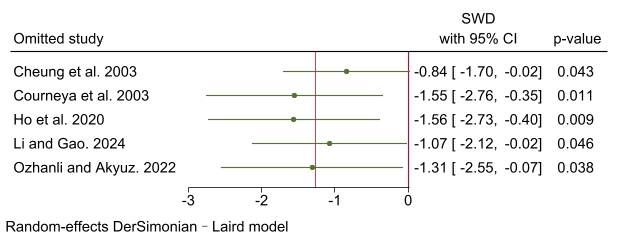
(a)


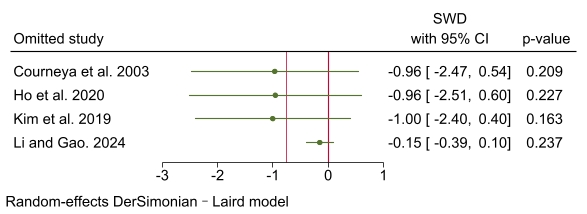


(b)


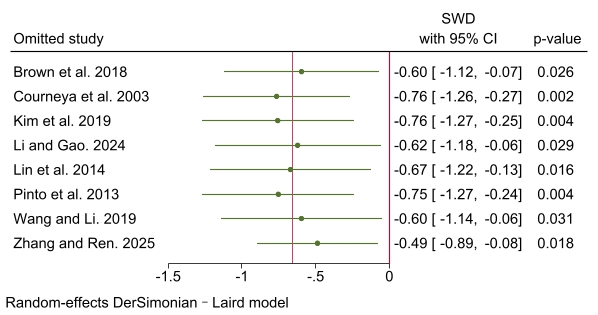
(c)


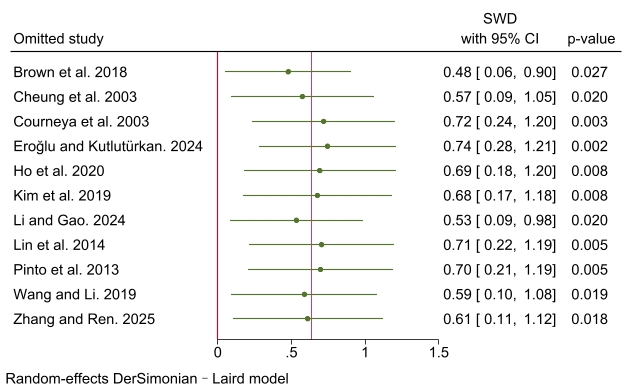


(d)

Figure S5. The sensitivity analyses for anxiety, depression, cancer-related fatigue, and quality of life. (a) anxiety; (b) depression; (c) cancer-related fatigue; (d) quality of life.


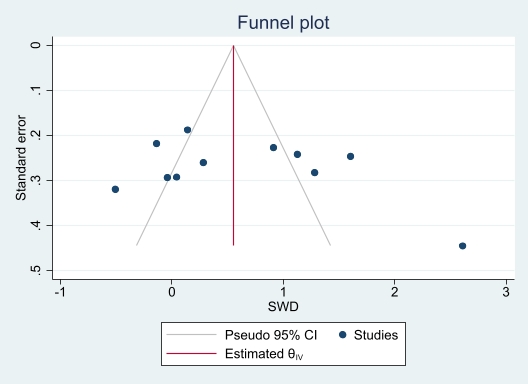


Figure S6. Funnel plot for quality of life.


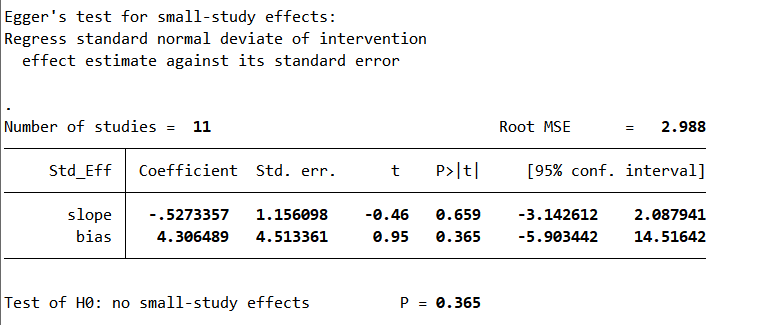
Figure S7. The result of Egger's test.

**Table S2.** GRADE summary of the quality of the evidence for the outcomes.

| Outcome | Quality assessment | | | | | No. of Participants  (studies) | Effect size (95% CI) | Quality of the evidence (GRADE) |
| --- | --- | --- | --- | --- | --- | --- | --- | --- |
|  | Risk of bias | Inconsistency | Indirectness | Imprecision | Other considerations |  |  |  |
| 1. Anxiety | Serious^a^ | Serious^b^ | No serious^c^ | No serious^d^ | None | 413 (5) | SMD = -1.26, [-2.24, -0.29] | ⨁⨁⭘⭘  Low |
| 1. Depression | Serious^a^ | Serious^b^ | No serious^c^ | Serious^e^ | None | 349 (4) | SMD = -0.76, [-1.81, 0.30] | ⨁⭘⭘⭘  Very low |
| 1. Cancer-related fatigue | Serious^a^ | Serious^b^ | No serious^c^ | No serious^d^ | None | 529 (8) | SMD = -0.66, [-1.14, -0.18] | ⨁⨁⭘⭘  Low |
| 1. Quality of life | Serious^a^ | Serious^b^ | No serious^c^ | No serious^d^ | None | 739 (11) | SMD = 0.64, [0.18, 1.10] | ⨁⨁⭘⭘  Low |

^a^ Most information is from studies rated as moderate or high risk of bias.

^b^ Heterogeneity in the I^2^ test >50%.

^c^ The home-based exercise is compared directly with the similar controls.

^d^ The 95% CI excludes pooled effect sizes that are not clinically significant.

^e^ The 95% CI includes pooled effect sizes that are not clinically significant.
